# Exploring Child-Parent Relationship Therapy -CPRT- Impact on Externalised Behaviours of Foster Children Experienced Complex Trauma: A Case Study

**DOI:** 10.1192/j.eurpsy.2024.1261

**Published:** 2024-08-27

**Authors:** S. Hatam, S. Moss, C. Cubillo, S. Parsafar, D. Berry

**Affiliations:** ^1^Faculty of Health; ^2^ Charles Darwin University; ^3^healing circle psychology, Darwin; ^4^interplay play therapy australia, Perth, Australia

## Abstract

**Introduction:**

Many foster children experience traumatic events that result in a wide range of disruptive behaviours, such as temper tantrums, superficially charming, no sincere remorse, and so forth. These problematic behaviours are challenging to the implementation of holistic therapeutic interventions.

**Objectives:**

The purpose of the current study is to explore the effectiveness of employing Child-Parent Relationship Therapy (CPRT) on externalised behaviours of a traumatised child at home and in social interactions.

**Methods:**

This study used a case study to explore the influence of CPRT on externalised behaviours of traumatised foster children. The case study focused on the externalised problematic behaviours of an Australian Aboriginal child fostered under long-term care at 18 months by a Caucasian family. The foster parent and the foster child received a 10-session structured CPRT across ten weeks. The child’s externalised behaviours were evaluated through the Child Behaviour Checklist (CBCL) form.

**Results:**

The findings describe the process of a 10-session structured CPRT with a foster child who exhibits externalised disruptive behaviours because of abandonment and complex trauma. The themes in the foster parent’s role and the play therapy approach relate to traumatic events and attachment issues. The results indicated a slight reduction in these behaviours. According to the follow-up interview, the child still showed aggressive behaviours in social interactions but not at home. Interviews with the foster parent indicated both the parent and child require additional support and further sessions of CPRT.

**Conclusions:**

This case study identified an improvement in externalised behaviours for foster children with experience of complex trauma and abandonment after a 10-session CPRT. Further research is required to explore the effectiveness of a longer-term session of CPRT alongside additional support services for foster parents.

**Disclosure of Interest:**

None Declared

